# Study on the spatial distribution characteristics of microbial communities and their environmental driving factors in Bing’an Reservoir after initial impoundment

**DOI:** 10.3389/fmicb.2026.1768906

**Published:** 2026-04-07

**Authors:** Lu Liu, Wenyi Zhang, Chunxiao Wang, Xiaobo Ge, Yunxiu Cao, Qian Zhang

**Affiliations:** 1 Power China Guiyang Engineering Corporation Limited, Guiyang, Guizhou, China; 2 School of Liquor and Food Engineering, Guizhou University, Guiyang, Guizhou, China

**Keywords:** Bing’an Reservoir, Chishui River, environmental driving factors, high-throughput sequencing, microbial communities, planktonic bacteria

## Abstract

After the initial impoundment of Bing’an Reservoir on the Chishui River, alterations are likely to occur in the structural composition and diversity of microbial communities. To unravel the microbial community diversity, community abundance, phylum composition, and spatial distribution characteristics within the reservoir, this study collected water samples from the surface, shallow, and middle layers. High-throughput sequencing technology targeting the bacterial 16S rRNA-encoding gene was employed to analyze the composition and diversity of microbial communities at different depths. The results demonstrated that the planktonic bacterial community in the reservoir was predominantly composed of prokaryotes (bacteria and archaea), with high microbial diversity and abundance. Among these, *Pseudomonas* and *Massilia* were the relatively dominant genera. The community structure and diversity of microorganisms were closely associated with water quality conditions. Results of variation partitioning analysis revealed that the impact of physicochemical factors on microbial community structure was significantly greater than that of geographical factors. Canonical correspondence analysis indicated that total phosphorus (TP), temperature (T), chemical oxygen demand (COD), pH, chlorophyll-a (Chl-a), ammonium nitrogen (NH_4_^+^-N), dissolved oxygen (DO), where the major driving forces shaping the microbial community structure. This study contributes to a better understanding of the ecosystem functions of Bing’an Reservoir, especially during the initial impoundment stage, and provides a scientific reference for the improvement and management of the water environment in the reservoir area.

## 1 Introduction

Microorganisms constitute an essential component of aquatic ecosystems. Aquatic plankton communities, which consist of microbial components (especially planktonic bacteria), zooplankton and phytoplankton, play an irreplaceable role in maintaining the dynamic stability of aquatic ecosystems. In particular, planktonic bacteria (Zhu et al., 2018; Liu et al., 2019) can assimilate nutrients derived from fish carcasses, fallen leaves and other organic debris, thereby preventing the occurrence of water eutrophication.

Bing’an Reservoir is located in Sanfo Village, Bing’an Township, Chishui City. It borders Xishui County of Guizhou Province to the southeast and shares boundaries with Gulin, Xuyong and Hejiang Counties of Sichuan Province to the northwest. The dam site is 383 km away from Guiyang, 240 km from Chongqing, and 350 km from Chengdu. The Bing’an River, where the reservoir is situated, is a first-class tributary on the left bank of the Chishui River within the Yangtze River Basin, with a catchment area of 62.3 km^2^ above the dam site. The reservoir has a total storage capacity of 21.442 million m^3^, a useful storage capacity of 19.556 million m^3^, and a dead storage capacity of 0.756 million m^3^. The project commenced construction in May 2018 and completed impoundment in December 2024. As a comprehensive water conservancy and livelihood project primarily for urban and rural water supply and secondarily for farmland irrigation, the reservoir can annually guarantee water supply for 160,000 residents in downtown Chishui and four surrounding towns, while meeting the irrigation needs of 1.15 km^2^ of cultivated land.

Before the dam construction, the Bing’an River section of the Chishui River was a natural fluvial ecosystem, where hydrological regimes were regulated by seasonal precipitation variations. The river featured unimpeded hydrological connectivity and active water exchange. Through long-term natural evolution, the microbial communities in this section developed a stable structure with high species richness and complementary ecological functions. Various types of microorganisms participated in key ecological processes such as organic matter decomposition and nutrient cycling via synergistic metabolism, jointly sustaining water cleanliness and ecological equilibrium. However, the completion of the dam and subsequent impoundment brought about fundamental transformations to the regional ecosystem: the original lotic habitats were converted into lentic reservoir habitats, resulting in a significant extension of hydraulic retention time; fish migration channels were blocked, disrupting their reproductive behaviors and population structures; the sediment transport process within the basin was altered, with substantial amounts of sediment depositing in the reservoir. This led to changes in the physical and chemical properties of the benthic environment, further affecting the habitat and reproduction of benthic organisms such as shrimp and shellfish. The combined effects of these multiple environmental disturbances on the aquatic ecosystem inevitably prompted adaptive changes in the structural composition, diversity, and functional traits of microbial communities. Critically, the reduced water mobility following reservoir impoundment caused the accumulation of terrestrially-sourced organic matter and organic matter derived from aquatic organism metabolism within the reservoir. This, in turn, altered the spatial distribution patterns of key physicochemical factors such as dissolved oxygen (DO) and nutrients (nitrogen and phosphorus), which are the core driving factors regulating the structure and functions of microbial communities (He et al., 2024).

Changes in microbial community structure are directly linked to key ecosystem functions such as material cycling efficiency and the self-purification capacity of reservoirs. The analysis of their spatial distribution characteristics thus serves as a crucial foundation for assessing the stability and health status of the reservoir ecosystem (Chen et al., 2022). During dam construction and the subsequent early impoundment phase, drastic alterations in hydrological conditions (e.g., increased water depth, reduced flow velocity, and prolonged water residence time) trigger profound shifts in physicochemical environments, which in turn drive significant succession in the composition and diversity of aquatic microbial communities (Liang et al., 2023; Wang et al., 2020). Previous studies have documented that early impoundment typically leads to increased nutrient accumulation and shifts from lotic (flowing water) to lentic (standing water) microbial taxa, fundamentally altering ecological processes (Peng et al., 2012). However, most existing research has focused on mature reservoirs or large river systems, with limited attention paid to the spatiotemporal dynamics of microbial communities during the initial impoundment stage of newly constructed reservoirs, especially regarding how vertical stratification (e.g., variations in temperature, DO, and nutrients) shapes microbial distribution patterns in the near-dam zone. This knowledge gap highlights the necessity of the present study to fill this void.

Therefore, a systematic investigation into the spatial distribution characteristics of aquatic microbial communities in Bing’an Reservoir during the initial impoundment period, together with an elucidation of their diversity, community composition, and key environmental drivers, is of great significance. It not only advances the mechanistic understanding of microbial community responses during the river-to-reservoir ecosystem transition induced by water conservancy projects, and enriches the available cases of microbial ecology in mountain reservoirs at the early impoundment stage.

It also provides a critical theoretical foundation and practical reference for developing scientific and rational water environment management strategies, optimizing ecological protection measures, and maintaining the stable operation of the reservoir ecosystem (Zhen-Jun et al., 2024; Newton et al., 2011; Nyirabuhoro et al., 2020).

## 2 Materials and methods

### 2.1 Sampling site setup and sample collection

At the time of this study, the reservoir was in the initial impoundment stage, with an unstable shoreline, poor traffic accessibility in some areas, and significant fluctuations in hydrological conditions during impoundment. Restricted by the actual on-site conditions, large-scale sampling with extensive site layout was objectively unfeasible at that time. In this experiment, the water from Bing’an Reservoir was selected as the research object. Surface, shallow, and middle water layers at different locations within the reservoir were sampled, with the sampling sites numbered W1, W2, and W3 in sequence. The sampling depths at each site were 0 m, 3 m, and 6 m, labeled as W1.0, W1.3, W1.6, W2.0, W2.3, W2.6, W3.0, W3.3, and W3.6, respectively, amounting to a total of 9 water samples.

**FIGURE 1:**
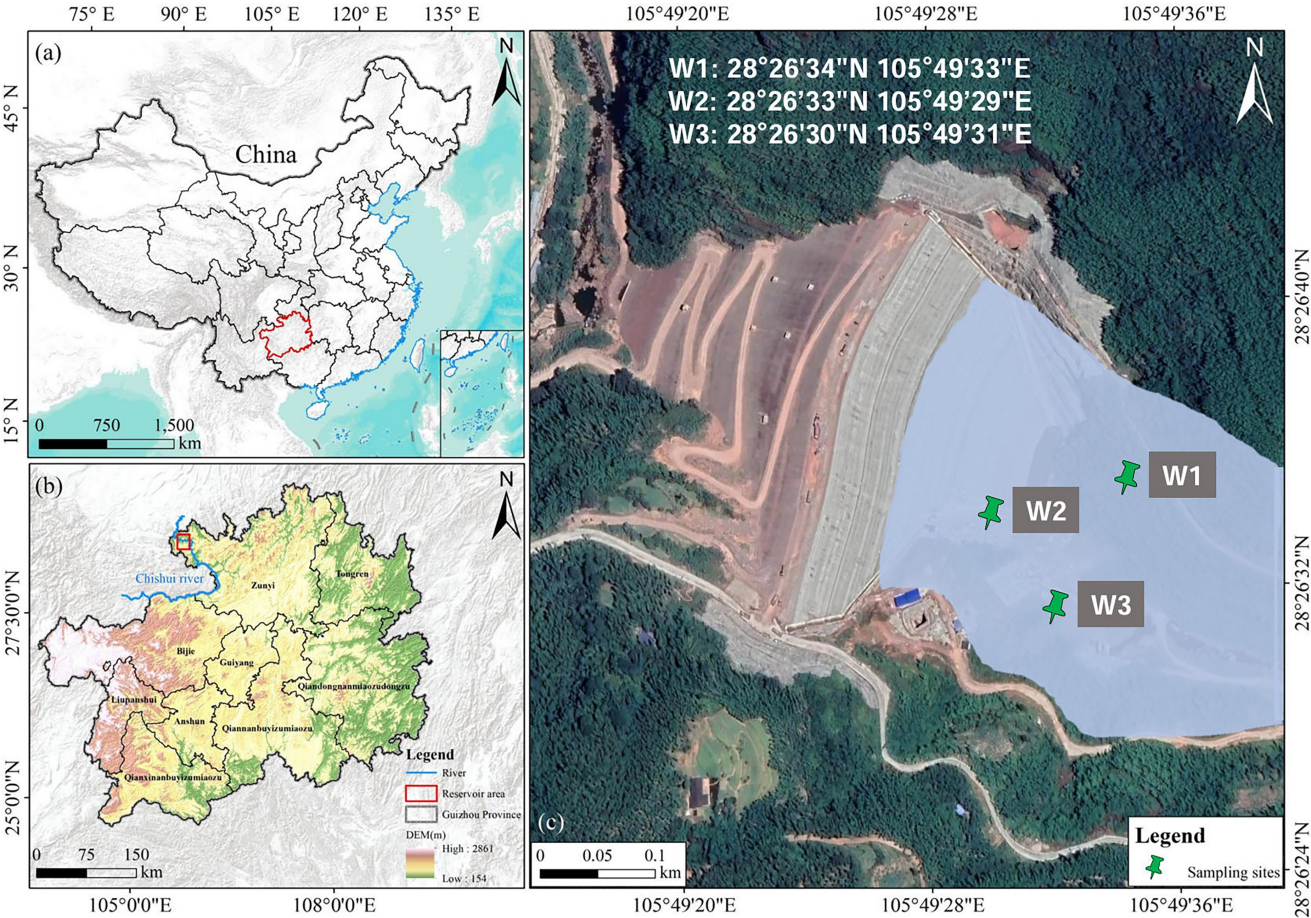
Location and sampling site distribution of the study area. **(a)** The location of Guizhou Province (red outline) within China, showing the regional geographic context at the national scale. **(b)** Topographic map of Guizhou Province, with the Chishui River basin highlighted (blue line) and the study reservoir area marked (red box), illustrating the watershed and terrain setting. **(c)** High-resolution satellite image of the reservoir, showing the locations of three sampling sites (W1, W2,and W3) with their precise geographic coordinates.

1.0 L glass water sampler was used for sample collection. The collected water samples were placed in polyethylene bottles and transported back to the laboratory for analysis and preservation. Under a sterile environment in the laboratory, the samples were filtered through a 0.22 μm pore-size membrane filter. After filtration, the membrane was placed in a cryotube with the addition of a DNA preservation solution and stored at −80°C. The survey scope, together with the details and layout of sampling sites, are presented in Figure 1.

### 2.2 Methods

High-throughput sequencing of the samples was performed on the Illumina platform. For the valid sequences obtained by sequencing, QIIME2 software was used for alignment against the Silva 138 database. With a 97% sequence similarity as the threshold, OTU clustering and taxonomic annotation were performed, and the composition of microbial communities at each taxonomic level was statistically analyzed. Subsequently, we carried out different testing methods in the laboratory and obtained the parameters of various water physical and chemical factors. The experimental procedures were divided into three steps: water sample DNA extraction, PCR amplification and high-throughput sequencing, and high-throughput data analysis (Lü et al., 2021), with the detailed procedures described as follows.

#### 2.2.1 Water sample DNA extraction

For each water sample, 600 mL of surface water was transferred into a sterilized beaker. Large-sized phytoplankton and zooplankton were removed by filtration through a 20 μm filter screen. The pretreated water sample was then filtered through a 0.22 μm sterile membrane filter to collect planktonic bacteria. Finally, the filter membrane was retrieved in an ultra-clean workbench, cut into pieces, and placed into a 50 mL pre-sterilized centrifuge tube.

Planktonic bacterial DNA was extracted using the Omega Water DNA Kit (Omega, USA). The concentration and purity of the collected planktonic bacterial DNA were determined with a NanoDrop ND-1000 micro-volume ultraviolet spectrophotometer (Wilmington, DE, USA).

#### 2.2.2 High-throughput sequencing and PCR product amplification

Using the total DNA extracted from the water samples as the template, the bacterial 16S rDNA sequences were amplified with the universal bacterial primers: 343F (TACGGRAGGCAGCAG) and 798R (AGGGTATCTAATCCT). The amplified products were analyzed by 1% agarose gel electrophoresis (Wang et al., 2016; Zwart et al., 2002), and then the PCR products were purified using a centrifugal column-type agarose gel extraction kit (Chen et al., 2002, 2022). Subsequently, high-throughput sequencing of the samples was performed on the Illumina NovaSeq platform.

#### 2.2.3 High-throughput data analysis

Data generated from sequencing on the Illumina NovaSeq platform were imported into Excel spreadsheets, followed by alpha diversity analysis of microorganisms. The corresponding Chao index (for estimating total species number), ACE index (for quantifying the number of bacterial species, genera, etc., in the sequencing results), Shannon index (for reflecting microbial diversity), and Simpson index (for reflecting microbial diversity and evenness) were calculated, respectively. On this basis, Origin software was used to perform statistical comparative analyses such as cluster analysis and variance partitioning analysis (VPA) on the species composition of these data.

#### 2.2.4 Methods for measuring water physicochemical parameters

The environmental indicators in this study mainly referred to the physicochemical indices of water, including pH, T (temperature), DO (dissolved oxygen), TN (total nitrogen), TP (total phosphorus), NO_3_^–^-N, NO_2_^–^-N, NH4^+^-N, ADP (algal degradation products), COD (chemical oxygen demand) and Chl-a. Among them, pH was measured *in situ* using a portable pH meter; T (temperature) was determined with a portable water temperature meter; DO (dissolved oxygen) was measured via the *in situ* electrode method with a portable dissolved oxygen meter; TN (total nitrogen) was assayed by the alkaline potassium persulfate digestion-ultraviolet spectrophotometry; TP (total phosphorus) was determined by the ammonium molybdate spectrophotometry; NO_3_^–^-N was measured using ultraviolet spectrophotometry; NO_2_^–^-N was assayed by the N-(1-naphthyl)-ethylenediamine spectrophotometry; NH4^+^-N was determined via the Nessler’s reagent spectrophotometry; ADP (algal degradation products) was characterized by algae-derived DOC (Dissolved Organic Carbon) and measured through the combustion oxidation-non-dispersive infrared absorption method; COD (chemical oxygen demand) was assayed by the potassium dichromate method; Chl-a (Chlorophyll a) was determined by spectrophotometry with acetone extraction. The geographical factors in this study included longitude and latitude, which were measured using a portable GPS receiver. After acquiring the aforementioned physicochemical indices of water and geographical factors, variation partitioning analysis (VPA) was performed using Origin software, with X1 (physicochemical factors) and X2 (geographical factors) set as explanatory variables, and Y (relative species abundance) as the response variable.

## 3 Results

### 3.1 Microbial community diversity index

**FIGURE 2:**
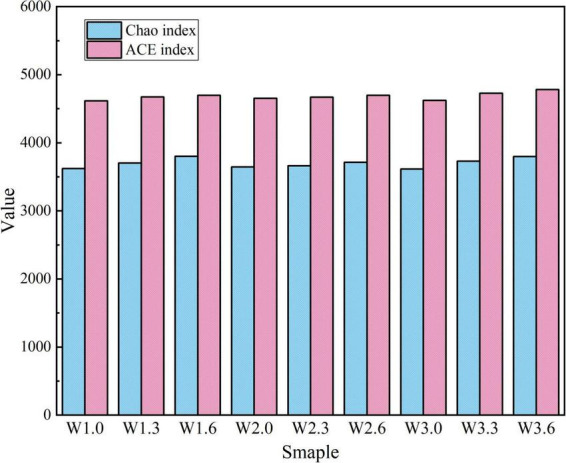
Analysis of Chao index and ACE index.

**FIGURE 3:**
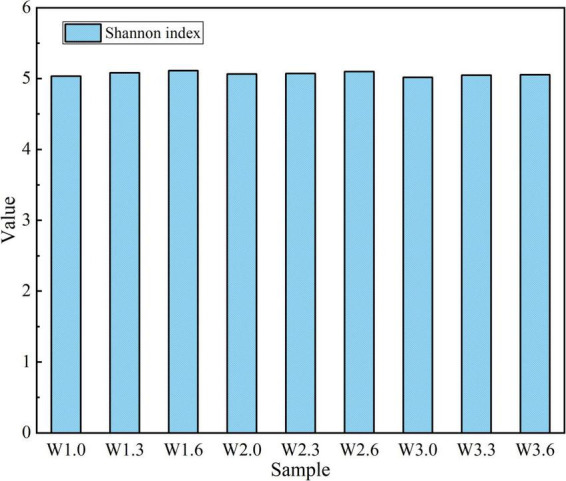
Analysis of Shannon index.

**FIGURE 4:**
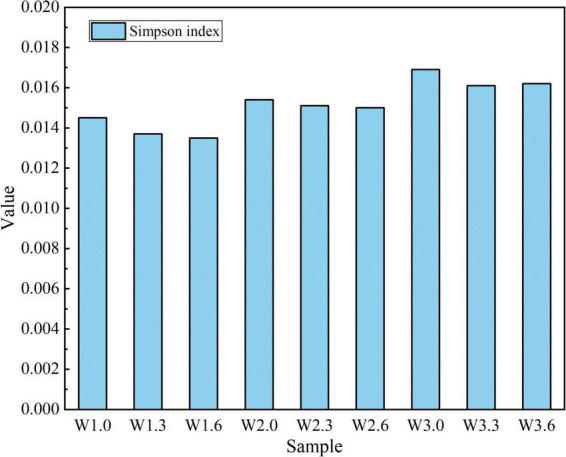
Analysis of Simpson index.

Microbial diversity indices, including the Chao, ACE, Shannon and Simpson indices, were determined for nine water samples collected from three sampling sites via the online analysis platform of Illumina Inc (Figures 2–4). As shown in Figure 2, the Chao index varied from 3,616 to 3,802 and the ACE index from 4,615 to 4,782 across all samples, indicating slight fluctuations in microbial species richness among different sampling sites and water layers with an overall high richness level. To assess the statistical significance of such inter-group variations, Kruskal–Wallis H tests were performed on the Chao and ACE indices, which revealed no significant differences among groups (Chao: H = 1.87, *P* = 0.973; ACE: H = 2.12, *P* = 0.969). These results suggested that the observed fluctuations in species richness were merely random variations rather than statistically significant differences.

As presented in Figure 3, the Shannon index of the water samples ranged from 5.018 to 5.112, and the Kruskal–Wallis H test confirmed no significant inter-group difference (H = 1.64, *P* = 0.983). This finding demonstrated that all samples exhibited a consistently high level of microbial diversity with stable species richness across different sampling sites and water layers.

As illustrated in Figure 4, the Simpson index of the samples varied from 0.0135 to 0.0169, with relatively low values overall. The Kruskal–Wallis H test showed no significant difference in the Simpson index among groups (H = 6.78, *P* = 0.564), indicating a generally low evenness of the microbial communities and the presence of a few dominant populations. Notably, this community characteristic was consistent across all sampling sites and water layers in the study area.

In addition, unweighted pair-group method with arithmetic mean (UPGMA) cluster analysis was conducted for the nine samples based on the Bray–Curtis distance matrix, and the Adonis test was further applied to verify the statistical significance of inter-group differences in microbial community structure. The results showed no significant clustering pattern of the microbial community structure among different sampling sites and water layers (*P* > 0.05), which further corroborated the conclusions derived from the above diversity index analyses.

### 3.2 Microbial community composition

**FIGURE 5:**
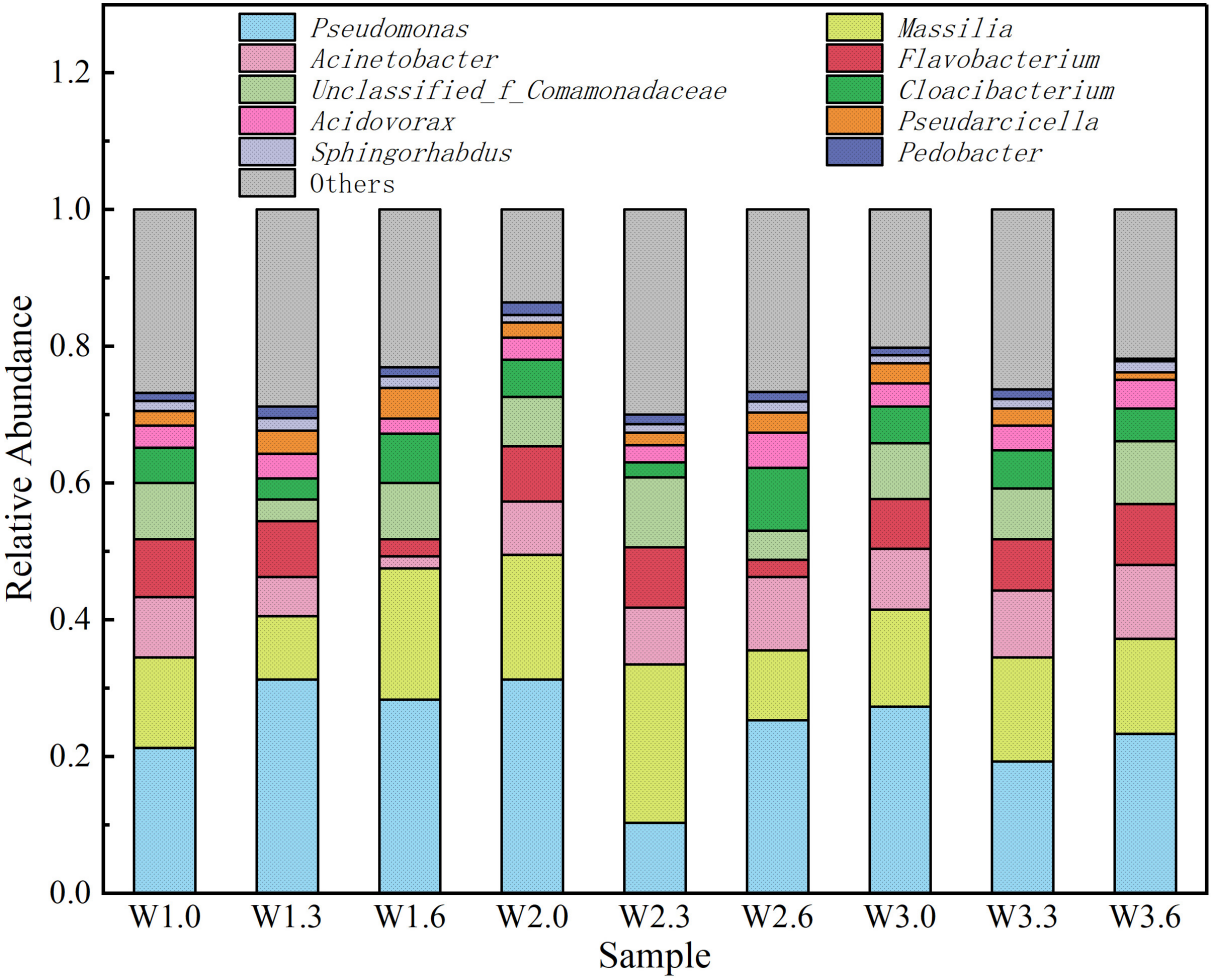
Relative abundance of different phyla.

**FIGURE 6:**
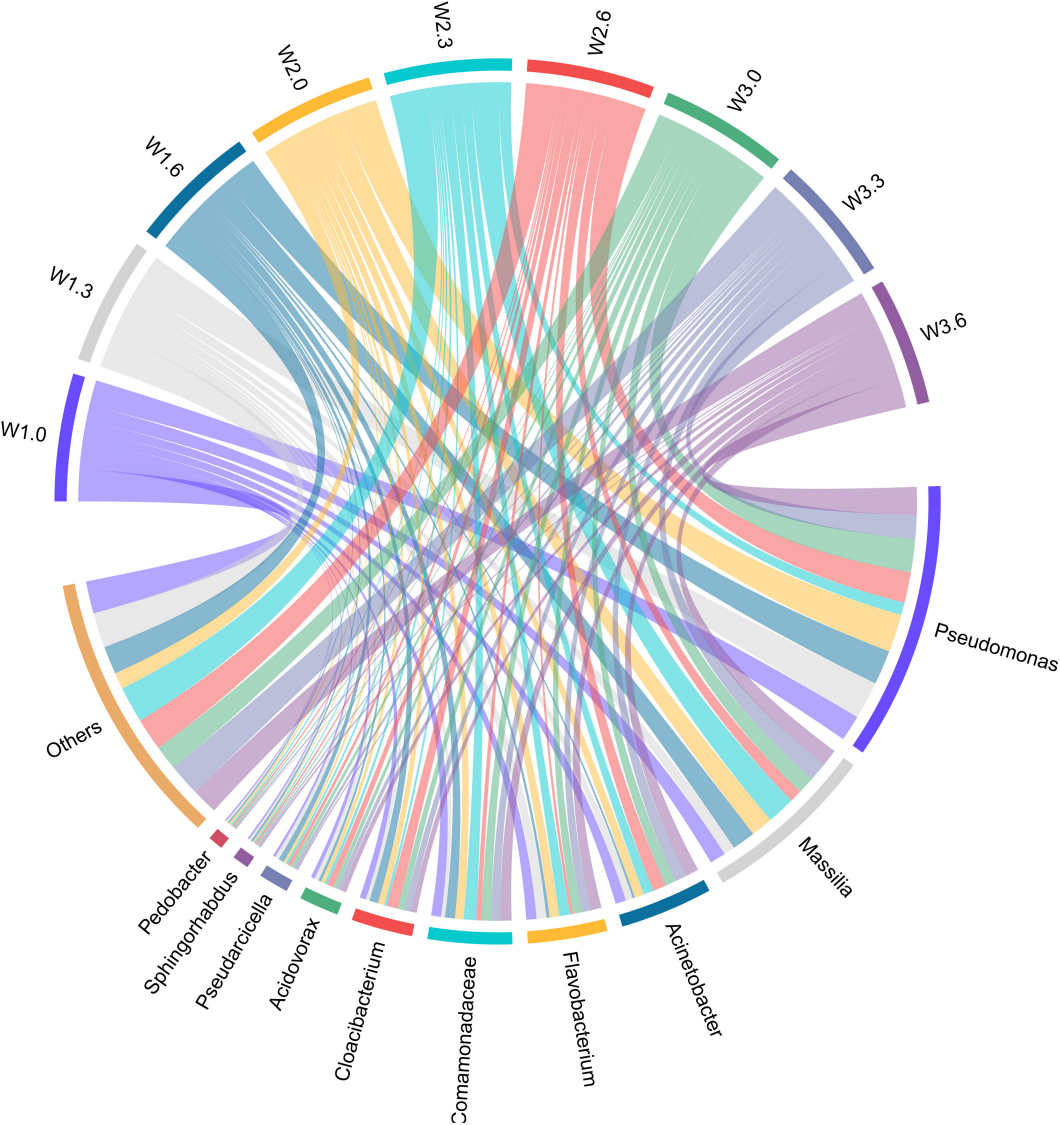
Chord diagram of data flow direction.

The relative abundance distribution and sample variation patterns of planktonic bacterial genera is presented in Figures 5, 6. High-throughput sequencing results revealed that the dominant bacterial genera detected in the water samples from Bing’an Reservoir included *Pseudomonas*, *Massilia*, *Acinetobacter*, *Flavobacterium*, and *Unclassified_f_Comamonadaceae*. Among these, *Pseudomonas* (*k_Bacteria*, *p_Proteobacteria*, *c_Gammaproteobacteria*, *o_Pseudomonadales*, *f_Pseudomonadaceae*, *g_Pseudomonas*, *s_Pseudomonas*) accounted for 10.3%–31.3% of the total abundance, while *Massilia* (*k_Bacteria*, *p_Proteobacteria*, *c_Betaproteobacteria*, *o_Burkholderiales*, *f_Comamonadacea*e, *g_Massilia*, *s_Massilia*) accounted for 9.2%–23.2%. Collectively, these two genera constituted 24.2% and 15.2% of the total bacterial community, respectively, making them the absolute dominant populations in the reservoir.

*Pseudomonas* is a genus of Gram-negative bacteria widely distributed in aquatic environments, with strong organic matter degradation capabilities that enable it to break down a variety of complex organic compounds (Chen et al., 2002). *Massilia* belongs to the family Comamonadaceae, order Burkholderiales, class Betaproteobacteria; most strains of this genus exhibit stress tolerance and pollutant degradation characteristics (Chen et al., 2022). The presence of these two dominant bacterial genera may be associated with the accumulation of organic matter and the increased demand for potential pollutant degradation during the initial impoundment stage of the reservoir, reflecting the adaptive response of the microbial community to changes in the reservoir’s ecological environment (Yang et al., 2019).

### 3.3 Spatial distribution of microbial communities

Analysis of differences in microbial community structure across different water layers indicated that the microbial composition of water samples from the surface layer (0 m), shallow layer (3 m) and middle layer (6 m) exhibited distinct vertical distribution characteristics.

In terms of species abundance, the Chao index and ACE index of bottom water samples (6 m) were generally higher than those of surface and shallow layer samples. For instance, both the Chao index (3,798) and ACE index (4,782) of sample W3.6 were significantly higher than those of the surface sample (W3.0: Chao = 3,616, ACE = 4,622) and shallow layer sample (W3.3: Chao = 3,732, ACE = 4,728) at the same sampling site. This might be related to the greater deposition of organic matter in the bottom water body, where abundant nutrients provide more sufficient growth substrates for microorganisms (Yang et al., 2019).

From the perspective of dominant population distribution, the abundance of *Pseudomonas* was higher in surface and shallow layer water samples; for example, the abundance of *Pseudomonas* reached 31.3% in samples W1.3 and W2.0. In contrast, the proportion of *Massilia* relatively increased in bottom water samples, with its abundance reaching 23.2% in sample W2.3. Such vertical distribution differences may be affected by environmental factors such as dissolved oxygen and light intensity. The surface water body is rich in dissolved oxygen, which is more suitable for the growth of aerobic *Pseudomonas*. The dissolved oxygen content in the bottom water body is relatively low, while some strains of *Massilia* have a certain tolerance to hypoxia, thus forming a relative dominance in the middle layer.

### 3.4 Relationship between microbial communities and water physicochemical factors

**FIGURE 7:**
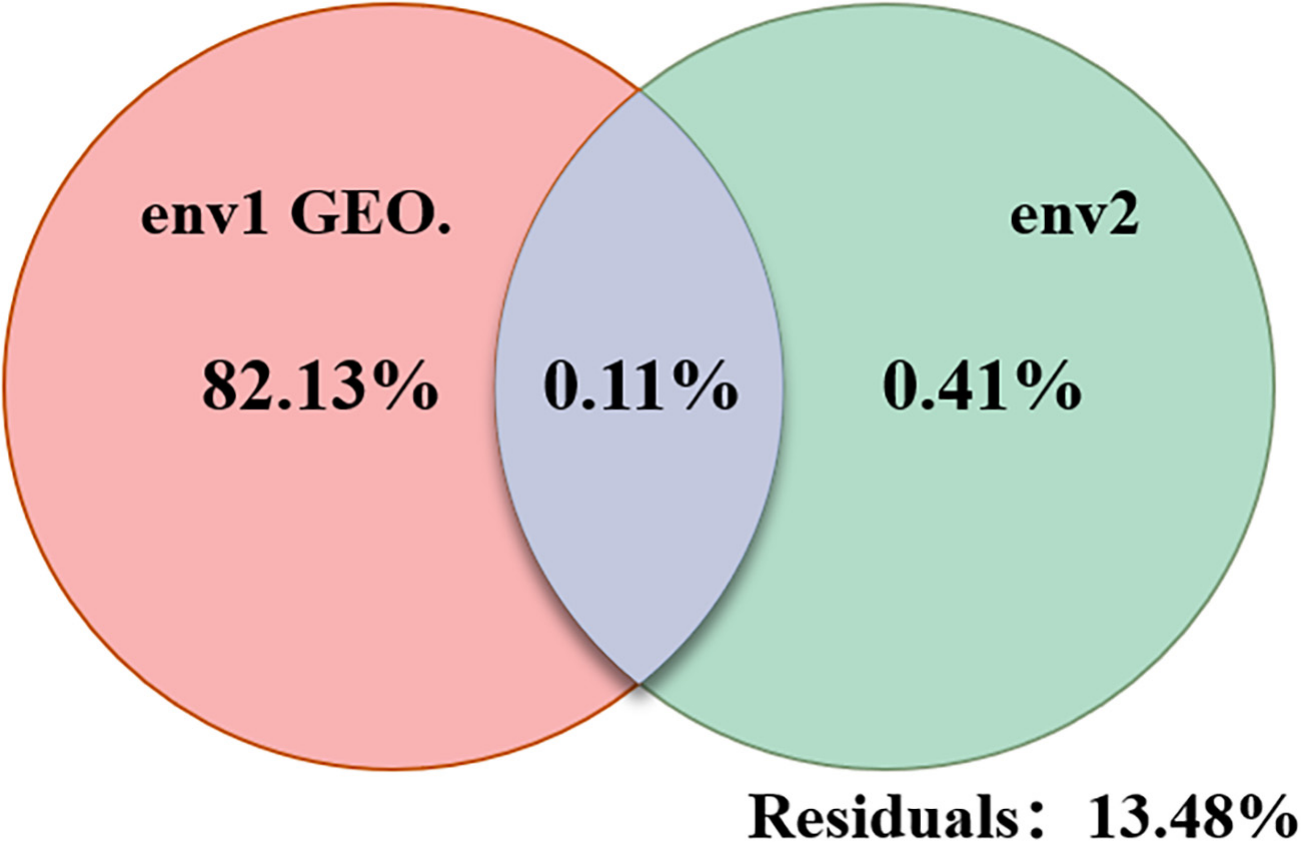
Results of variance partitioning analysis.

The purpose of variation partitioning analysis (VPA) is to determine the explanatory proportion of specified environmental factors to changes in community structure (Lindström et al., 2005; Wang et al., 2024; Zhao et al., 2021; Jiang et al., 2023). The analysis results are presented in Figure 7, which show that the individual explanatory power of the first group, envfit1, including environmental factors such as pH, T (temperature), DO (dissolved oxygen), TN (total nitrogen), TP (total phosphorus), NO_3_^–^-N, NO_2_^–^-N, NH_4_^+^-N, ADP (algal degradation products), COD (chemical oxygen demand), and Chl-a, reached 82.13%. The individual explanatory power of the second group, envfit2, including geographical factors such as longitude and latitude, was only 0.41%, while the joint explanatory power of the two groups was 0.11%. The above results indicate that the selected environmental factors, both physicochemical and geographical, have significant effects on the distribution of bacterial species in Bing’an Reservoir. However, it is worth noting that the first group of physicochemical factors has a significantly higher impact on the distribution of microbial community structure than the second group.

**FIGURE 8:**
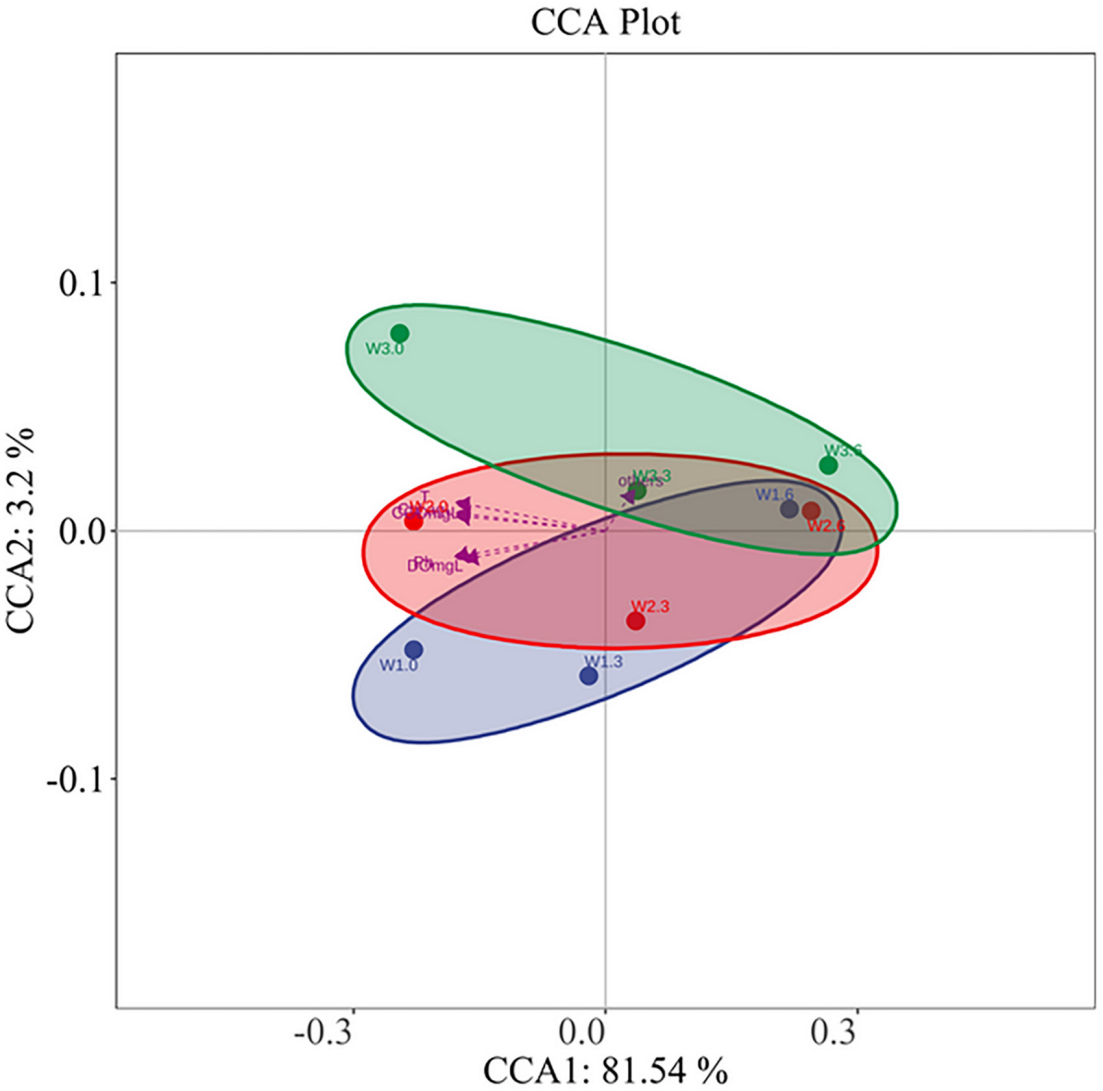
Canonical correspondence analysis (CCA) results.

The results of canonical correspondence analysis (CCA) reflect the relationship between bacterial communities and environmental factors, as shown in Figure 8. These results suggest that there was significant collinearity among most of these environmental factors. According to the variation partitioning analysis (VPA), pH, T, DO, TN, TP, NO_3_^–^-N, NO_2_^–^-N, NH_4_^+^-N, ADP, COD, Chl-a, longitude, and latitude all exerted an influence on the distribution of bacterial communities in Bing’an Reservoir (Ansari et al., 2022; Ni et al., 2015; Feng et al., 2023; Yang et al., 2023).

The length of the arrow represents the magnitude of the correlation. It can be clearly seen from Figure 8 that T, TP, COD, and SD had a relatively strong correlation with the distribution of bacterial communities. The envfit function was used to test the significance of each environmental factor. The results of the CCA envfit analysis of the relationship between bacteria and environmental factors in Bing’an Reservoir are presented in Figure 8. The environmental factors affecting the bacterial community distribution in Bing’an Reservoir were ranked in the following order of importance: T, TP, COD, DO, Chl-a and pH (*P* = 0.0005) etc.

Given that this study focused on a riverine water area, the distribution of bacterial communities was mainly affected by external environmental factors. Through CCA and VPA analyses, this study found that longitude and latitude had a minor impact on the community structure in Bing’an Reservoir, whereas TP, T, COD, pH, Chl-a, NH_4_^+^-N and DO were the dominant influencing factors.

## 4 Discussion

During the initial impoundment of Bing’an Reservoir, the planktonic microbial community exhibited a distinct structural signature of high species richness, elevated diversity, and low evenness—an inherent trait tightly linked to the transitional nature of hydrologically disturbed reservoir ecosystems. This structural characteristic is a common ecological phenomenon in reservoir ecosystems undergoing initial impoundment, as dam construction and water storage drastically alter the original riverine hydrological regime, leading to profound changes in the composition and distribution of planktonic microbial communities (Liang et al., 2023). Impoundment-induced reduced flow velocity facilitated the sedimentation of allochthonous (terrestrially derived) and autochthonous (*in situ* produced) organic matter, forming a nutrient-replete microenvironment with ample carbon and nitrogen substrates. This nutrient-rich condition provides sufficient energy and material sources for microbial growth and reproduction, thereby fueling microbial proliferation, which is corroborated by the high Chao and ACE indices—well-validated proxies for species richness in microbial ecology. Concurrently, drastic environmental perturbations (water level elevation, altered hydrological regime, and concomitant physicochemical shifts such as changes in pH, dissolved oxygen, and nutrient concentration) exerted intense selective pressure on the planktonic microbial community. Under such stress, *Pseudomonas* and *Massilia*—genera renowned for metabolic versatility and robust adaptability—emerged as dominant taxa. Their competitive edge in resource acquisition (e.g., efficient utilization of complex organic matter) and stress resilience reduced community evenness, reflecting a typical succession trajectory in disturbed aquatic ecosystems where resilient taxa dominate, which is consistent with the findings of a study on the initial impoundment stage of the Xiluodu Reservoir (Wang et al., 2010).

The ecological functions of *Pseudomonas* and *Massilia* highlight the reservoir’s material cycling potential during initial impoundment. Both genera efficiently decompose complex organic matter (including refractory organic pollutants) and mediate core biogeochemical cycles (e.g., carbon mineralization, nitrogen transformation) in freshwater habitats (Silby et al., 2011). Specifically, *Pseudomonas* can secrete a variety of extracellular enzymes to degrade lignin, cellulose, and other refractory organic substances, while *Massilia* plays a crucial role in ammonification, nitrification, and denitrification processes, jointly maintaining the nutrient balance of the reservoir ecosystem. Their proliferation sustains water quality stability by mitigating organic pollution and regulating nutrient dynamics, consistent with prior reservoir studies (Chen et al., 2002; Yang et al., 2019). Nevertheless, continuous organic matter accumulation in bottom water poses notable ecological risks: intensified decomposition depletes hypolimnetic dissolved oxygen (DO), and prolonged hypoxia enhances denitrification, disrupting nitrogen balance and potentially increasing nitrous oxide emissions or ammonia accumulation—both impairing reservoir health. A previous study on the Miyun Reservoir has demonstrated that long-term hypoxic conditions in bottom water can significantly increase ammonia nitrogen concentration and alter the structure of microbial communities, further exacerbating water quality degradation (Beaulieu et al., 2011). We propose prioritizing optimized hydrological regulation (e.g., increased water exchange frequency, reasonable water level regulation) to enhance bottom-water DO penetration, alleviate hypoxia, and foster a functionally balanced microbial community, thereby ensuring the stable operation of the Bing’an Reservoir ecosystem.

To assess conclusion generalizability and guide future research, inherent limitations—stemming from initial impoundment’s transitional properties and objective constraints—must be acknowledged. First, the study was confined to this transient stage, where unsteady environmental dynamics (water level, flow velocity, nutrient load) differ fundamentally from stable operation. Thus, observed community patterns and environmental drivers only reflect stage-specific characteristics, not the full disturbance-to-stability succession or stable-phase dynamics. This is a common limitation in short-term studies focusing on the initial impoundment stage of reservoirs, as the ecological system during this period is in a state of continuous change and adaptation (Liang et al., 2023). Second, the narrow impoundment time window (several months) precluded cross-seasonal sampling, leaving seasonal impacts on community assembly, functional traits, and adaptation strategies unexplored—critical given freshwater microbes’ sensitivity to seasonal temperature, precipitation, and nutrient pulses, which have been shown to significantly alter microbial community structure and functional potential in other freshwater ecosystems (Wang et al., 2010).

Furthermore, sample size (9 spatial/vertical sites) and data integrity are constrained. Complex hydrological conditions during impoundment prevented high-density sampling and replication, potentially obscuring fine-scale spatial patterns such as microhabitat differences in microbial distribution between near-shore and offshore areas. Early storage equipment malfunctions rendered some raw data (unscreened species matrices, continuous environmental monitoring) irretrievable, limiting cross-validation via RDA or Mantel test and in-depth analysis of microbe-environment interactions. Methodologically, reliance on high-throughput sequencing alone (without metagenomics/transcriptomics) failed to link “community structure-functional traits-ecological processes.” As reported in relevant studies, amplicon sequencing alone can only characterize microbial community structure, but cannot effectively reflect the functional potential and actual metabolic activity of the community (Silby et al., 2011). Additionally, irreproducible impoundment conditions, small sample size, and data gaps precluded quantifying multi-factor interactions via SEM, restricting driving mechanism analysis to correlational rather than causal insights, which limits the depth of our understanding of microbial community assembly mechanisms during initial impoundment.

Future research should address these limitations: (1) conduct long-term cross-stage monitoring (initial impoundment to stable operation) with seasonal sampling to characterize community responses to succession and seasonality, which will help clarify the dynamic changes of microbial communities throughout the entire life cycle of the reservoir; (2) optimize sampling/data storage, increase density/replication, and establish comprehensive archiving for multi-method validation, thereby improving the reliability and representativeness of research data; (3) integrate high-resolution physicochemical monitoring and advanced molecular tools (e.g., metagenomics, transcriptomics) to simultaneously characterize community structure and function, using RDA/Mantel test to deepen environmental driver analysis and construct a “structure-function-environment” framework. This approach will provide robust scientific support for Bing’an Reservoir’s ecological management from initial disturbance to sustainable operation, and also enrich the theoretical system of microbial ecology in hydrologically disturbed reservoir ecosystems (Beaulieu et al., 2011).

## 5 Conclusion

This study investigated the composition, spatial distribution, and driving factors of planktonic bacterial communities in Bing’an Reservoir during the initial impoundment stage using 16S rRNA high-throughput sequencing. The planktonic bacterial community showed high species richness and diversity but low evenness, with *Pseudomonas* and *Massilia* as the absolute dominant genera. Distinct vertical distribution patterns were observed: bacterial richness was higher in middle water layers, whereas *Pseudomonas* was more abundant in surface and middle layers and *Massilia* was enriched in bottom layers.

Total phosphorus, water temperature, COD, pH, chlorophyll-a, ammonium nitrogen, and dissolved oxygen were identified as the major environmental drivers shaping microbial community structure, while geographical factors had little influence. These results clarify the spatial distribution and assembly mechanisms of planktonic bacteria during early impoundment, providing a theoretical basis for water environment management and ecological protection of mountain reservoirs under river-to-reservoir transformation.

## Data Availability

The datasets presented in this article are not readily available because of the legal regulations and institutional policies of the authors’ university and enterprise. Requests to access the datasets should be directed to Lu Liu, powerchinaliulu@163.com.

## References

[B1] Ansari M. I. Calleja M. L. Silva L. Viegas M. Ngugi D. K. Huete-Stauffer T. M. et al. (2022). High-frequency variability of bacterioplankton in response to environmental drivers in red sea coastal waters. *Front. Microbiol.* 13:780530. 10.3389/fmicb.2022.780530 35432231 PMC9009512

[B2] Beaulieu J. J. Tank J. L. Hamilton S. K. Wollheim W. M. Hall R. O. Jr. Mulholland P. J. (2011). Nitrous oxide emission from denitrification in stream and river networks. *Proc. Nati. Acad. Sci. U.S.A.* 108 214–219. 10.1073/pnas.1011464108 21173258 PMC3017147

[B3] Chen C. J. Zhang H. Q. Wang Y. Q. Yu X. L. Wang J. F. Shen Y. L. (2002). [Characteristics of microbial community in each compartment of ABR ANAMMOX reactor based on high-throughput sequencing]. *Huan Jing Ke Xue* 37 2652–2658. 10.13227/j.hjkx.2016.07.031 29964475

[B4] Chen Z. J. Liu Y. Q. Li Y. Y. Lin L. A. Zheng B. H. Ji M. F. et al. (2022). The seasonal patterns, ecological function and assembly processes of bacterioplankton communities in the Danjiangkou Reservoir, China. *Front. Microbiol.* 13:884765. 10.3389/fmicb.2022.884765 35783417 PMC9240478

[B5] Feng J. Zhou L. Zhao X. Chen J. Li Z. Liu Y. et al. (2023). Evaluation of environmental factors and microbial community structure in an important drinking-water reservoir across seasons. *Front. Microbiol.* 14:1091818. 10.3389/fmicb.2023.1091818 36865780 PMC9971975

[B6] He F. Zarfl C. Tockner K. Olden J. D. Campos Z. Muniz F. et al. (2024). Hydropower impacts on riverine biodiversity. *Nat. Rev. Earth Environ.* 5 755–772. 10.1038/s43017-024-00596-0

[B7] Jiang X. Liu Y. Zhou R. Sun T. Cao J. An S. et al. (2023). Cascade dams altered taxonomic and functional composition of bacterioplankton community at the regional scale. *Front. Microbiol.* 14:1291464. 10.3389/fmicb.2023.1291464 37954247 PMC10634544

[B8] Liang S. Zhang F. Li R. Sun H. Feng J. Chen Z. et al. (2023). Field investigation on the change process of microbial community structure in large-deep reservoir during the initial impoundment. *J. Environ. Manage.* 338:117827. 10.1016/j.jenvman.2023.117827 37023606

[B9] Lindström E. S. Agterveld M. P. K.-V. Zwart G. (2005). Distribution of typical freshwater bacterial groups is associated with pH, temperature, and lake water retention time. *Appl. Environ. Microbiol.* 71 8201–8206. 10.1128/AEM.71.12.8201-8206.2005 16332803 PMC1317352

[B10] Liu M. Liu L. Chen H. Yu Z. Yang J. R. Xue Y. et al. (2019). Community dynamics of free-living and particle-attached bacteria following a reservoir Microcystis bloom. *Sci. Total Environ.* 660 501–511. 10.1016/j.scitotenv.2018.12.414 30640117

[B11] Lü X. B. Wu Y. C. Chen L. Q. Liu M. Q. Yang F. Wang M. M. et al. (2021). Characteristics of the bacterioplankton community and their relationships with water quality in Chishui River Basin. *Acta Sci. Circumstantiae* 41, 4596–4605. 10.13671/j.hjkxxb.2021.0125

[B12] Newton R. J. Jones S. E. Eiler A. McMahon K. D. Bertilsson S. (2011). A guide to the natural history of freshwater lake bacteria. *Microbiol. Mol. Biol. Rev.* 75 14–49. 10.1128/MMBR.00028-10 21372319 PMC3063352

[B13] Ni Z. Huang X. Zhang X. (2015). Picoplankton and virioplankton abundance and community structure in Pearl River Estuary and Daya Bay, South China. *J. Environ. Sci.* 32 146–154. 10.1016/j.jes.2014.12.019 26040741

[B14] Nyirabuhoro P. Liu M. Xiao P. Liu L. Yu Z. Wang L. et al. (2020). Seasonal variability of conditionally rare taxa in the water column bacterioplankton community of subtropical reservoirs in China. *Microb. Ecol.* 80 14–26. 10.1007/s00248-019-01458-9 31836929

[B15] Peng Q. Xie B. Yuan Q. Huang Z. T. Wang W. T. (2012). [Preliminary study on the changes of bacterial community structure in Qingcaosha Reservoir during water storage period]. *Huan Jing Ke Xue* 33, 3634–3640. 10.1007/s11783-011-0280-z 23233999

[B16] Silby M. W. Winstanley C. Godfrey S. A. Levy S. B. Jackson R. W. (2011). *Pseudomonas* genomes: Diverse and adaptable. *FEMS Microbiol. Rev.* 35:21361996. 10.1111/j.1574-6976.2011.00269.x 21361996

[B17] Wang H. Shen Z. Niu J. He Y. Hong Q. Wang Y. (2010). Functional bacteria as potential indicators of water quality in Three Gorges Reservoir, China. *Environ. Monit. Assess.* 163 607–617. 10.1007/s10661-009-0863-3 19333770

[B18] Wang J. Peng J. F. Song Y. H. Yuan L. Shi G. (2016). Seasonal changes of microbial community distribution in sediments of Hun River. *Res. Environ. Sci.* 29:9. 10.13198/j.issn.1001-6929.2016.02.06

[B19] Wang Q. Yu J. Li X. Zhang Y. Zhang J. Wang J. et al. (2024). Seasonal and anthropogenic influences on bacterioplankton communities: Ecological impacts in the coastal waters of Qinhuangdao, Northern China. *Front. Microbiol.* 15:1431548. 10.3389/fmicb.2024.1431548 38962120 PMC11220261

[B20] Wang Y. Lu L. Hong Y. Wu J. Zhu G. Ye F. et al. (2020). Divergent responses of taxonomic and predicted functional profiles of bacterioplankton to reservoir impoundment. *Environ. Res.* 182:109083. 10.1016/j.envres.2019.109083 31901627

[B21] Yang E.-D. Cui D.-X. Wang W.-Y. (2019). Research progress on the genus *Massilia*. *Microbiol. China* 46 1537–1548. 10.13344/j.microbiol.china.180573

[B22] Yang Y. Chen Y. Li Z. Zhang Y. Lu L. (2023). Microbial community and soil enzyme activities driving microbial metabolic efficiency patterns in riparian soils of the Three Gorges Reservoir. *Front. Microbiol.* 14:1108025. 10.3389/fmicb.2023.1108025 37180230 PMC10171112

[B23] Zhao J. Peng W. Ding M. Nie M. Huang G. (2021). Effect of water chemistry, land use patterns, and geographic distances on the spatial distribution of bacterioplankton communities in an anthropogenically disturbed riverine ecosystem. *Front. Microbiol.* 12:633993. 10.3389/fmicb.2021.633993 34025599 PMC8138559

[B24] Zhen-Jun L. Qian-Qian Z. Rui-Lian G. Yan Z. Ji-Cheng Y. Zhen-Bing W. U. et al. (2024). Composition of microbial community structure in water and sediments of xia’shan reservoir in Shandong Province. *Acta Hydrobiologica Sinica*. 48, 2081–2091. 10.7541/2025.2024.0260

[B25] Zhu J. Hong Y. Zada S. Hu Z. Wang H. (2018). Spatial Variability and Co-acclimation of Phytoplankton and Bacterioplankton Communities in the Pearl River Estuary, China. *Front. Microbiol.* 9:2503. 10.3389/fmicb.2018.02503 30405565 PMC6206238

[B26] Zwart G. Crump B. C. Agteveld M. P. K. V. Hagen F. Han S.-K. (2002). Typical freshwater bacteria: An analysis of available 16S rRNA gene sequences from plankton of lakes and rivers. *Aquat. Microb. Ecol.* 28 141–155. 10.3354/ame028141

